# Genetic regulation of newborn telomere length is mediated and modified by DNA methylation

**DOI:** 10.3389/fgene.2022.934277

**Published:** 2022-10-04

**Authors:** Congrong Wang, Rossella Alfano, Brigitte Reimann, Janneke Hogervorst, Mariona Bustamante, Immaculata De Vivo, Michelle Plusquin, Tim S. Nawrot, Dries S. Martens

**Affiliations:** ^1^ Centre for Environmental Sciences, Hasselt University, Hasselt, Belgium; ^2^ ISGlobal, Barcelona, Spain; ^3^ Universitat Pompeu Fabra, Barcelona, Spain; ^4^ CIBER de Epidemiología y Salud Pública, Madrid, Spain; ^5^ Center for Genomic Regulation, Barcelona Institute of Science and Technology, Barcelona, Spain; ^6^ Channing Division of Network Medicine, Department of Medicine, Brigham and Women’s Hospital and Harvard Medical School, Boston, MA, United States; ^7^ Program in Genetic Epidemiology and Statistical Genetics, Harvard School of Public Health, Boston, MA, United States; ^8^ Department of Public Health and Primary Care, Leuven University, Leuven, Belgium

**Keywords:** newborn, telomere, ageing, genetic variants, DNA methylation, mQTL, mediation and effect modification

## Abstract

Telomere length at birth determines later life telomere length and potentially predicts ageing-related diseases. However, the genetic and epigenetic settings of telomere length in newborns have not been analyzed. In addition, no study yet has reported how the interplay between genetic variants and genome-wide cytosine methylation explains the variation in early-life telomere length. In this study based on 281 mother-newborn pairs from the ENVIR*ON*AGE birth cohort, telomere length and whole-genome DNA methylation were assessed in cord blood and 26 candidate single nucleotide polymorphism related to ageing or telomere length were genotyped. We identified three genetic variants associated with cord blood telomere length and 57 *cis* methylation quantitative trait loci (*cis*-mQTLs) of which 22 mQTLs confirmed previous findings and 35 were newly identified. Five SNPs were found to have significant indirect effects on cord blood telomere length *via* the mediating CpGs. The association between rs911874 (*SOD2*) and newborn telomere length was modified by nearby DNA methylation indicated by a significant statistical interaction. Our results suggest that DNA methylation in *cis* might have a mediation or modification effect on the genetic difference in newborn telomere length. This novel approach warrants future follow-up studies that are needed to further confirm and extend these findings.

## 1 Introduction

Telomeres are ribonucleoprotein complexes that cap the end of eukaryotic chromosomes and function to protect genomic stability. In normal somatic cells, telomere length declines with cell divisions until it reaches a critical length, which triggers cellular senescence and finally leads to subsequent programmed cell death. Therefore, telomere length determines the replicative capacity and the lifespan of a cell (O'Sullivan and Karlseder, 2010; Allsopp et al., 1992; Codd et al., 2013). At the level of an individual, somatic cell telomeres are the longest at birth and normal telomere attrition occurs throughout the life course (Frenck et al., 1998). At birth and among same-aged individuals, considerable variability exists in telomere length (Slagboom et al., 1994), which is partly due to heritability and partly to potential accelerated telomere shortening induced by environmental and cellular stressors (Aubert and Lansdorp, 2008). Telomere length tracks over different life stages (Bijnens et al., 2017; Martens et al., 2021), and shorter telomere lengths in adulthood is associated with age-related diseases (Zhao et al., 2013; Blackburn et al., 2015) and mortality (Wang et al., 2018).

The genetic determinants of adult telomere length have been widely investigated. Genome-wide association studies (GWAS) have identified single nucleotide polymorphisms (SNPs) related to telomere length (Levy et al., 2010; Prescott et al., 2011; Mangino et al., 2012; Codd et al., 2013; Liu et al., 2014; Mitchell et al., 2014; Mangino et al., 2015; Codd et al., 2021) and lifespan (Lunetta et al., 2007; TenNapel et al., 2014). Previous twin studies have indicated that heritable factors could account for up to 78% of the variation in telomere length (Slagboom et al., 1994; Hannon et al., 2018). Besides genetic variants related to telomere length, epigenetic signatures (DNA cytosine methylation) of telomere length (Buxton et al., 2014; Lee et al., 2019) and telomere attrition (Wang et al., 2021a) have been described. However, little is known on the potential interplay between genetic and epigenetic regulations of telomere length. Gene-specific allelic variations are closely associated with differences in DNA methylation in proximal regions of this allelic variation, as shown by published DNA methylation quantitative trait (mQTL) analyses (Gaunt et al., 2016; Volkov et al., 2016; Ciuculete et al., 2017). Given the role of the epigenome in regulating gene expression (Moore et al., 2013), the potential mediation effect of DNA methylation has been studied on the genetic liability to a specific phenotype (Howe et al., 2019).

In this study, within a subset of the ongoing prospective birth cohort, ENVIRonmental influence *ON* early AGEing (ENVIR*ON*AGE), we aimed to investigate the combined genetic-epigenetic regulation of newborn telomere length, assessing biological ageing-related SNPs and DNA methylation. The following hypotheses were tested: 1) genetic variants in adult biological ageing-related genes are associated with newborn telomere length, 2) these genetic variants affect the DNA methylation within the same or nearby genes, which mediate the genetic association with newborn telomere length, and 3) the association between a genetic variant and newborn telomere length is modified by DNA methylation levels in nearby regions.

## 2 Materials and methods

### 2.1 Study population

This study took place within the context of the ongoing prospective ENVIR*ON*AGE birth cohort (Janssen et al., 2017) initiated in 2010. This cohort study has been approved by the ethical committee of Hasselt University and East-Limburg Hospital (Genk, Belgium), and is carried out according to the Declaration of Helsinki. Between February 2010 and May 2015, 885 mothers were recruited upon arrival in the hospital for delivery, and were considered eligible if they were able to fill out a questionnaire in Dutch. Written informed consent was obtained from the participating mothers and questionnaires were filled out by the mothers to collect information on lifestyles and socioeconomic status. Information on newborns’ sex, birth weight and gestational age, and maternal age and parity was collected from medical records in the hospital. The date of conception was estimated based on the first day of the mother’s last menstrual period in combination with the first ultrasonographic examination. Maternal body mass index (BMI) was determined as the ratio of the maternal weight to the squared maternal height measured at the first antenatal visit (weeks 7-9 of pregnancy). The ethnicity of a newborn was categorized as European if at least two grandparents were Europeans and classified as non-European otherwise. The educational level of the mothers was coded as “0” if they did not obtain any diploma, as “1” if they obtained at highest a high school diploma, as “2” if the highest diploma obtained was from a 3-year college and as “3” if they obtained a 4-year college or university degree. Maternal smoking status was classified into “never smoker”, “former smoker” (quit smoking before pregnancy), or “smoker” (continued smoking during pregnancy). Mothers were regarded to have had pregnancy complications if they had any of the following conditions: gestational diabetes, hypertension, hyper- or hypothyroidism, infectious disease, preeclampsia, vaginal bleeding, phenylketonuria and allergy or asthma during pregnancy. The ENVIR*ON*AGE birth cohort is representative of all births in Flanders with regard to maternal age and education, parity, newborn’s sex, ethnicity, and birth weight (Janssen et al., 2017). Among the recruited mother-newborn pairs, 855 had telomere length measurement at birth, of which 365 were also profiled on genome-wide DNA methylation. After excluding the newborns without SNP genotyping and one mother missing information on smoking status, 281 mother-newborn pairs were included in the current study (Supplementary Figure S1).

### 2.2 Biological sample collection and DNA extraction

Cord blood was collected immediately after delivery in BD Vacutainer® plastic whole blood tubes with spray-coated K2EDTA (BD, Franklin Lakes, NJ, USA). Samples were centrifuged at 3,200rpm for 15 min. Plasma was removed and the remainder of the samples with the buffy coats was stored at -80 °C until further analysis.

Fetal placental biopsies (1–2 cm^3^) were taken at the fetal side directly underneath the chorioamniotic membrane, at approximately 4 cm from the umbilical cord. Contamination by chorioamniotic membrane was avoided by visual examination and dissection. Histological examination confirmed that the fetal placental biopsies mainly contained cytotrophoblasts and syncytiotrophoblast differentiated from trophoblasts.

Cord blood and placental DNA were extracted using the QIAamp DNA mini kit (Qiagen, Inc., Venlo, Netherlands). DNA purity and concentration were verified using a Nanodrop 1,000 spectrophotometer (Isogen, Life Science, Belgium). DNA was considered pure when the A260/280 was greater than 1.80 and A260/230 was greater than 2.0. DNA integrity was assessed with agarose gel electrophoresis.

### 2.3 SNP selection and genotyping

Sixteen SNPs reported to be related to telomere length in previous GWAS (Prescott et al., 2011; Mangino et al., 2012; Codd et al., 2013; Liu et al., 2014; Mitchell et al., 2014; Mangino et al., 2015) and 18 SNPs shown to be associated with lifespan in a GWAS (Lunetta et al., 2007) or in a candidate gene study (TenNapel et al., 2014) were selected *a priori* for genotyping in placental DNA. SNP genotyping was conducted using the Biotrove OpenArray SNP Genotyping Platform at the Dana Farber/Harvard Cancer Center Genotyping and Genetics for Population Sciences Facility. After excluding SNPs with a SNP call rate lower than 95% and samples with a sample call rate lower than 90%, genotype imputation was performed with R package statgenGWAS (version 1.0.7.1) (van Rossum et al., 2020) based on the Beagle software (Browning and Browning, 2016). The genotyping data were further filtered to remove SNPs with a minor allele frequency (MAF) < 0.01 and a Hardy-Weinberg equilibrium chi-squared test *p*-value < 0.05. After preprocessing, 26 candidate SNPs related to telomere length or ageing traits were included in this study. The selection of SNPs and the original publications are shown in Supplementary Table S1. The pairwise linkage-disequilibrium (LD) was evaluated by plotting the allelic correlation *r* ^2^ matrix in a heatmap, using R package LDheatmap (version 1.0–4). SNP genotype frequency data can be found in the European Variation Archive with accession numbers PRJEB53351 (project) and ERZ11081188 (analyses).

### 2.4 DNA methylation measurement and data processing

Cord blood DNA samples were bisulphite-converted, amplified and hybridized to the Illumina HumanMethylationEPIC Bead-Chip array (Illumina, San Diego, CA, USA), at the service lab GenomeScan (Leiden, Netherlands), for 377 cord blood samples from ENVIR*ON*AGE. Array measurements were scanned using an Illumina iScan and the data quality was assessed using the R script MethylAid. DNA methylation data were preprocessed using the minfi package (version 1.38.0) in R (Aryee et al., 2014). Briefly, a probe was excluded when the probe call rate was lower than 95% based on a detection *p*-value larger than 10e-16 (Lehne et al., 2015). One sample was excluded because of the sample call rate lower than 99%, and four other samples were excluded due to discordant sex prediction using shinyMethyll (version 1.28.0) (Fortin et al., 2014a). Methylation data were normalized using functional normalization (Fortin et al., 2014b). For each CpG locus, the methylation level was expressed as M value calculated using the signal intensity from methylated probes and unmethylated probes (Du et al., 2010). Preprocessing resulted in a DNA methylation dataset with 857,898 CpGs. Missingness was imputed by K-nearest neighbor (KNN) imputation (K = 10) and technical confounding effects (batch and position) were removed from the M-value using an empirical Bayes method (Johnson et al., 2007). Subsequently, we trimmed the data per CpG for outliers defined as lower than three inter-quarter-ranges (IQR) below the first quartile, or higher than three IQRs above the third quartile. CpG probes were filtered to exclude CpGs on X and Y chromosomes, those known to be common SNPs and those having cross-reactivity with multiple genomic locations (Pidsley et al., 2016). 787,264 CpGs remained available for the statistical analyses.

Based on the DNA methylation data, blood cell proportions (nucleated red blood cells, granulocytes, monocytes, natural killer cells, B cells, CD4^+^ T cells, and CD8^+^ T cells) in the cord blood samples were estimated using Bakulski algorithm (Bakulski et al., 2016).

### 2.5 Average relative telomere length measurement and data processing

Average relative telomere length was measured in cord blood samples in triplicate using a previously described quantitative, real-time polymerase chain reaction (qPCR) protocol (Martens et al., 2016). Detailed specifications of the assay are provided in the supplementary Method. Telomere length was measured as the ratio of telomere copy number to single-copy gene number (T/S) relative to the average T/S ratio of the entire sample set. The inter-assay intra-class correlation coefficient (ICC) (TELOMERE RESEARCH NETWORK, 2020) was 0.936 (95% CI: 0.808–0.969) and the intra-assay ICC was 0.952 (95% CI: 0.947–0.956). A coefficient of variation (CV) of 6.4% was achieved within triplicates of the T/S ratios.

### 2.6 Statistical analyses

The statistical analyses workflow is shown in Supplementary Figure S2. In each step, the models were adjusted for a set of *a priori* selected covariates: newborn’s sex, gestational age, ethnicity and birth weight, maternal pregnancy complications, pre-pregnancy BMI, parity, education level and smoking status and paternal age, as well as cell type heterogeneity estimated from the DNA methylation data.

#### 2.6.1 Step 1: Genotype-telomere length association analyses

In the first step, cord blood telomere length was regressed on each SNP in a multiple regression model. Dominant coding was applied, where the genotype at each SNP was classified into either of the two categories: major homozygote and heterozygote/minor homozygote. To ensure robustness of the results, we additionally applied an additive coding based on the number of minor alleles, which is a commonly used SNP coding method in the literature (Tam et al., 2019). Statistical significance of a model term was defined as having a nominal *p*-value lower than 0.05.

#### 2.6.2 Step 2: Genotype-methylation association analysis

Each of the selected 26 SNPs was analyzed against CpG sites available in cis (within ±0.5 Mb from the SNP), by fitting multiple regression models of the M-value of each CpG on the dominant-coded or additive-coded SNPs. The R package limma (version 3.48.1) was used for model fitting and inference. Statistical significance was determined under multiple testing control by Bonferroni correction at a level of 0.05. The identified significant SNP-CpG pairs were compared with a published methylation quantitative trait loci (mQTL) database (www.mqtldb.org), specifying the timepoint as “newborns” and the distance defining *cis*-mQTLs as 500 kb Gaunt et al., 2016.

#### 2.6.3 Step 3: Mediation analyses

The CpGs involved in the significant mQTL were assessed for their association with cord blood telomere length. Using the CpGs that were identified both in mQTL and in association with cord blood telomere length, a path model was constructed for the mediating effect of the CpGs on the SNP-telomere length association, based on the following assumptions: 1) genotypes determined at the very beginning of life precede both the change in DNA methylation and the shortening of telomere length, and 2) genotypes are less likely to be changed than DNA methylation and telomere length shortening, which are regulated through more complex pathways. Therefore, it was assumed that the genetic variants at a certain SNP altered the methylation level at certain CpGs, which subsequently led to a change in newborn telomere length. Mediation analyses were performed with the R package mediation (version 4.5.0). Tingley et al., (2014). Dominant coding was used for SNPs. The direct effect (DE), the indirect effect (IE) and the total effect (TE) were estimated, and the proportion of mediated was estimated in case that the DE and ID were in the same direction. Statistical inference was performed using bootstrap percentiles with 1,000 bootstrap samples and nominal significance level of 0.05 was adopted for each effect.

#### 2.6.4 Step 4: Genotype-methylation interaction

The *cis*-interaction between CpG and SNP was assessed by including a statistical interaction term (SNP*CpG) in the model of cord blood telomere length. The model is stated as:

$$cordTL=\beta_{0}+\beta_{1}SNP+\beta_{2}\text{CpG}+\beta_{12}\text{SNP}\;*\;\text{CpG}+C^{T}\gamma +\varepsilon$$ (1)

where C denotes all covariates. The coefficient $\beta_{12}$ provides the estimate of the interaction effect. Additive coded SNPs were used to avoid singular fit or convergence problem. Significant statistical interactions were determined with Bonferroni correction at a level of 0.05. Interactions consisting of a CpG-SNP pair that had a significant association in the mQTL analysis were dropped to avoid multicollinearity, because the independence of the genotype and the methylation level could not be assumed in those cases.

## 3 Results

### 3.1 Characteristics of the study population

Characteristics of the study population, comprising 281 mother-newborn pairs, are summarized in Table 1. The numbers of newborn girls and boys were balanced, and the majority (93.6%) of the newborns were of European origin. The gestational age ranged between 29 and 41 weeks, and around 6.0% were born preterm (before week 37). The newborns had an average birth weight of 3.38 ± 0.48 kg and around 2.8% were of low birth weight (lower than 2.5 kg). Mothers had a mean age of 30.11 ± 4.17 years and a pre-pregnancy body-mass index (BMI) of 24.03 ± 4.21 kg/m2. Among all the mothers, 65.8% had received a higher education. Thirty-three mothers (11.7%) reported having smoked during pregnancy, whereas most mothers (73.0%) never smoked. More than half of the newborns were from the first pregnancy of the mother (51.6%). In total 48 mothers (17.1%) experienced one or more pregnancy complications.

**TABLE 1:** Characteristics of the study population (N=281).

Characteristics	N (%) or mean ± SD
***Newborns***	
*Sex*	
Female	139 (49.5%)
Male	142 (50.5%)
*Ethnicity*	
European	263 (93.6%)
Non-European	18 (6.4%)
*Birth weight (kg)*	3.38 ± 0.48
*Gestational age (weeks)*	39.03 ± 1.64
*Cord blood telomere length (T/S ratio)*	1.03 ± 0.20
***Mothers***	
*Educational level*	
No diploma	16 (5.7%)
High school diploma	80 (28.5%)
A 3-year college	148 (52.6%)
A 4-year college or university	37 (13.2%)
*Smoking status*	
Never smoker	205 (73.0%)
Former smoker	43 (15.3%)
Smoker	33 (11.7%)
*Parity*	
Primiparous	145 (51.6%)
Secundiparous	105 (37.4%)
Multiparous	31 (11.0%)
*With pregnancy complications*	48 (17.1%)
*Age at delivery (years)*	30.11 ± 4.17
*Pre-pregnancy BMI* (*kg/m* ^*2*^)	24.03 ± 4.21
***Fathers***	
*Age at child birth (years)*	32.48 ± 4.99

### 3.2 SNPs in association with cord blood telomere length

The model estimates under dominant coding and additive coding, respectively, are shown in Table 2. Without correction for multiple testing, the results with both coding methods showed weak associations. Three SNPs, rs9419958 (*OBFC1*), rs9420907 (*OBFC1*) and rs17653722 (*KRT80*), had estimates significantly different from zero in the dominant coding model. Rs9419958 (*OBFC1*) was confirmed in the additive coding model, while the other two SNPs, rs9420907 (*OBFC1*) and rs17653722 (*KRT80*), were only borderline-significant under additive coding.

**TABLE 2:** Association (nominal *p*-value) between SNPs and newborn telomere length^a^.

SNP (major/minor allele)	Position	Gene	Dominant coding^b^	Additive coding^c^
rs1475398 (G/C)	chr1:65517574	*LEPR*	-0.032 (0.20)	-0.028 (0.16)
rs1343981 (A/G)	chr1:65579645	*LEPR*	-0.014 (0.58)	-0.016 (0.42)
rs10493379 (G/A)	chr1:65580244	*LEPR*	-0.013 (0.61)	-0.016 (0.44)
rs6669117 (T/C)	chr1:65595389	*LEPR*	6.1e-5 (1.00)	-0.007 (0.69)
rs4452212 (G/A)	chr2:136258421	*CXCR4*	-0.040 (0.20)	-0.023 (0.20)
rs10496799 (T/C)	chr2:139261401	*NXPH2*	0.005 (0.83)	0.022 (0.28)
rs11125529 (C/A)	chr2:54248729	*ACYP2*	-0.002 (0.95)	-0.008 (0.75)
rs10936599 (C/T)	chr3:169774313	*TERC*	0.006 (0.80)	0.007 (0.75)
rs16847897 (G/C)	chr3:169850328	*TERC*	-0.001 (0.96)	-0.006 (0.78)
rs40184 (C/T)	chr5:1394962	*DAT1*	-0.029 (0.29)	-0.022 (0.19)
rs2841505 (T/G)	chr6:13571363	*SIRT5*	-0.004 (0.87)	-0.018 (0.37)
rs911847 (G/A)	chr6:159647936	*SOD2*	-0.008 (0.75)	0.003 (0.88)
rs3757354 (C/T)	chr6:16235386	*MYLIP*	0.022 (0.39)	0.031 (0.17)
rs2371208 (G/T)	chr7:82708543	*-*	-0.021 (0.39)	-0.021 (0.32)
rs9419958 (C/T)	chr10:103916188	*OBFC1*	**0.067 (0.01)**	**0.054 (0.03)**
rs9420907 (A/C)	chr10:103916707	*OBFC1*	**0.058 (0.03)**	0.047 (0.06)
rs511744 (C/T)	chr11:219089	*SIRT3*	-0.030 (0.21)	-0.033 (0.08)
rs17653722 (G/T)	chr12:52193734	*KRT80*	**-0.063 (0.01)**	-0.044 (0.05)
rs4764600 (C/G)	chr12:6492814	*GAPDH*	0.031 (0.21)	0.015 (0.41)
rs4570625 (G/T)	chr12:71938143	*TPH2*	0.017 (0.52)	0.013 (0.53)
rs1386494 (C/T)	chr12:71958763	*TPH2*	0.022 (0.43)	0.027 (0.29)
rs2535913 (G/A)	chr14:72948525	*DCAF4*	-0.037 (0.13)	-0.013 (0.50)
rs3027234 (C/T)	chr17:8232774	*CTC1*	0.009 (0.73)	0.016 (0.46)
rs412658 (C/T)	chr19:22176638	*ZNF676*	0.004 (0.86)	-0.010 (0.59)
rs107251 (C/T)	chr19:4176088	*SIRT6*	0.002 (0.94)	-0.001 (0.97)
rs755017 (A/G)	chr20:63790269	*RTEL1*	-0.010 (0.72)	-0.010 (0.69)

a Models were adjusted for newborn sex, gestational age, ethnicity, birth weight, maternal medical conditions during pregnancy, pre-pregnancy BMI, parity, education level, smoking status, paternal age and estimated cell type heterogeneity. Estimates in bold are significant at a 5% significance level.

b Dominant coding: a genotype was classified as major homozygote or heterozygote/minor homozygote.

c Additive coding: a genotype was coded as the number of minor alleles.

The 26 SNPs were not completely independent, as suggested by Supplementary Figure S3, in which three clusters of SNPs were detected by their pairwise linkage disequilibrium. Highly correlated SNPs (*r* ^2^ > 0.5), such as rs1475398 (*LEPR*), rs10493379 (*LEPR*) and rs1343981 (*LEPR*), rs9419958 (*OBFC1*) and rs9420907 (*OBFC1*), behaved consistently in their association with cord blood telomere length in terms of the effect size, direction and effect significance.

### 3.3 DNA methylation quantitative loci

The number of CpGs tested on each SNP is summarized in Supplementary Table S2. Among the 26 SNPs, rs755017 (*RTEL1*) had no CpGs within the 0.5 Mb neighborhood. In total, the association was tested on 12,194 SNP-CpG pairs. There were 57 *cis*-mQTL identified from both the dominant models and the additive models under Bonferroni correction (Supplementary Table S3). Matching our results with the external mQTL database mQTLdb (http://www.mqtldb.org/), (Gaunt et al., 2016) 22 out of the 57 SNP-CpG pairs were confirmed with the same direction and comparable effect size. The top association identified in the current study was between rs4764600 (*GAPDH*) and cg07142400 (*NCAPD2*; *MRPL51*), which confirmed the findings in mQTLdb, where one more copy of the minor allele G at rs4764600 was associated with more than 0.50 increase in the M-value at cg07142400 (*NCAPD2*; *MRPL51*). Most of the SNPs were associated with multiple CpGs that were not located in the same gene. SNP rs3027234 (*CTC1*) had the largest number of associated CpGs and the involved twelve CpGs mapped to seven genes. The SNP with the second largest number of associated CpGs was rs4764600 (*GAPDH*), with the nine CpGs distributed over five genes. On the other hand, five CpGs were associated with more than one SNP in other genes and all the other CpGs were only associated with one SNP.

### 3.4 DNA methylation mediates the genetic difference in cord blood telomere length

**FIGURE 1:**
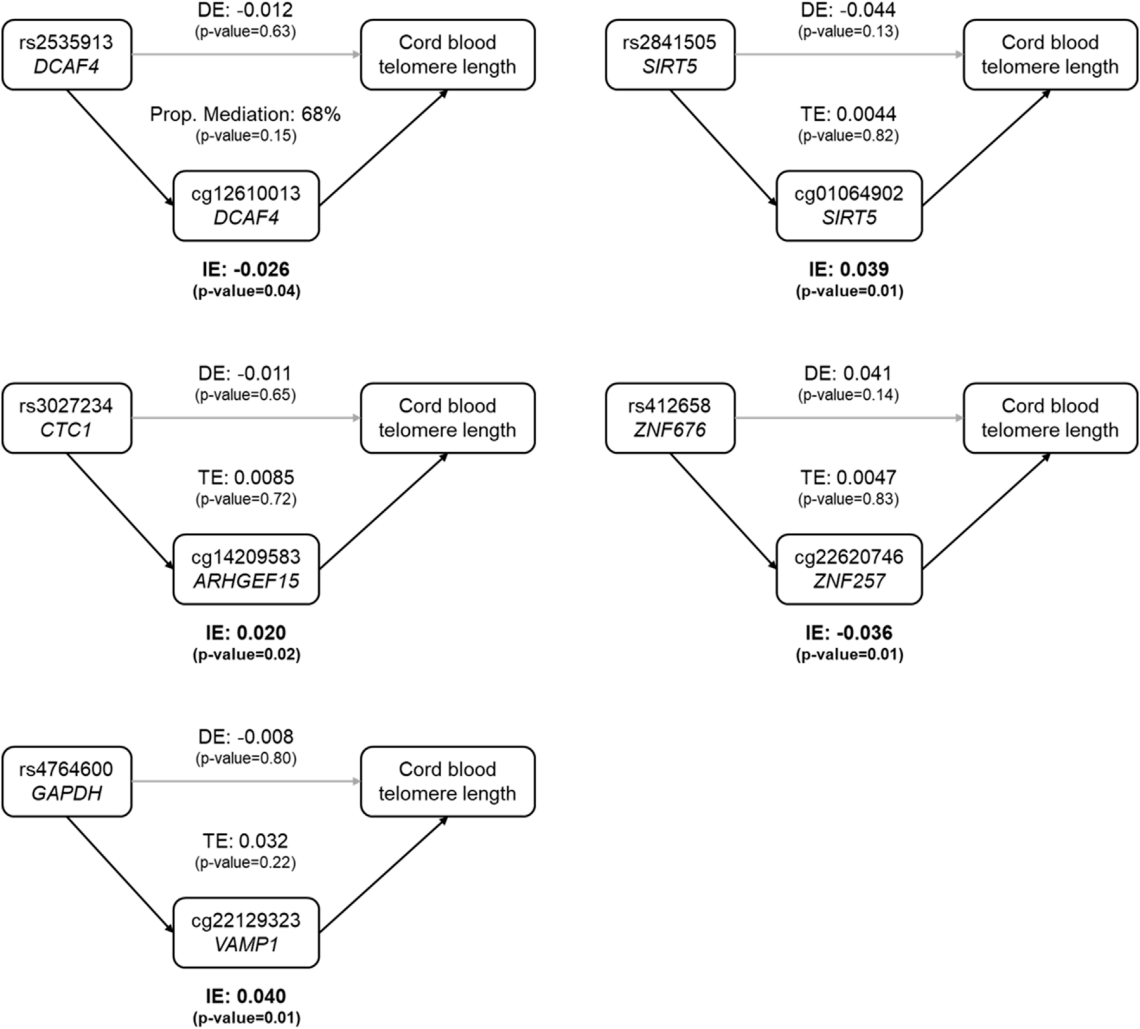
Estimated proportion of the association between SNP genotypes and cord blood telomere length mediated by the DNA methylation level at a CpG. In each sub-figure, the estimate of the indirect effect (IE) *via* the CpG, and the estimate of the direct effect (DE) of the SNP on cord blood telomere length are displayed. For the first SNP-CpG pair (rs2535913 and cg12610013, top left), where IE and DE were in the same direction, the proportion of mediation (IE/DE + IE) was shown. In the other 4 mediation relationships, IE and DE had opposite directions, and therefore only the total effect (TE) was shown. Grey arrows stood for insignificant associations, while black arrows denoted the statistically significant ones (at a nominal significance level of 0.05). Dominant coding of SNPs was used and the mediation model was adjusted for newborn’s sex, gestational age, ethnicity and birth weight, maternal pregnancy complications, pre-pregnancy BMI, parity, education level and smoking status, paternal age, and estimated cell type heterogeneity.

Among the 48 CpGs involved in the 57 mQTLs, five CpGs were found to be associated with cord blood telomere length at a nominal significance level (Supplementary Table S4). These five SNP-CpG pairs were used to construct mediation models as shown in Figure 1, where all SNPs’ indirect effect was mediated by only one CpG. All five indirect effects were significant at the 5% significance level, and none of the direct effects or the total effects was significant (Figure 1 and Supplementary Table S5). For rs2535913 (*DCAF4*), the direct and indirect effects were in the same direction and the proportion mediated was 68% (*p*-value = 0.15, Figure 1). For all the other SNPs, the direct effects were in the opposite direction of the corresponding indirect effects.

### 3.5 DNA methylation modified genetic difference in cord blood telomere length

A SNP-CpG interaction effect was identified between rs911874 (*SOD2*) and cg24223887 (gene unknown) under Bonferroni correction (β = -0.330, *p*-value = 6.2e-5), indicating that the higher the methylation level at cg24223887 (gene unknown), the weaker the association between the number of A allele at rs911847 (*SOD2*) and cord blood telomere length. Within the same model, the SNP rs911874 (*SOD2*) main effect was significant under the Bonferroni correction for SNP main effects, which has not been identified in the candidate SNP association analysis without SNP-CpG interactions (Table 2). The number of A allele was associated with a 1.186 T/S ratio (*p*-value = 6.4e-5) increase in cord blood telomere length. The CpG main effect of cg24223887 was nominally significant (0.263, *p*-value = 8.2e-04).

## 4 Discussion

Telomere length is a biomarker of ageing and early-life telomere length relates to later life telomere length and may underlie ageing-related diseases. How the interplay between genetic and epigenetic factors is related to newborn telomere length remains to be understood. In the present study, we showed that DNA methylation on specific CpGs in the near genomic distance to biological age-related genetic variants might mediate the genetic regulation of newborn telomere length. In addition, the genetic difference in newborn telomere length might be altered by the DNA methylation level in a nearby region.

In the candidate gene association analysis of newborn telomere length, we studied 26 telomere length- or lifespan-related candidate SNPs that were previously described in adults. Although these candidate SNPs were identified in previous studies mostly by GWAS under stringent control of type I error, we did not observe clear associations with newborn telomere length under stringent control of type I error. While limited statistical power due to sample size could be one explanation, a previous candidate gene study of mother-newborn pairs was similarly unable to reproduce the GWAS findings of cord blood telomere length for three of the SNPs used in the current study (rs11125529 (*ACYP2*), rs10936599 (*TERC*) and rs755017 (*RTEL1*)) (Weng et al., 2016). This might be explained by the fact that these variants are involved in telomere length maintenance against risk factors that arise from external stimuli in later life, rather than in the programming of telomere length at birth. In the current study, SNPs in *OBFC1* (rs9419958 and rs9420907) and in *KRT80* (rs17653722) were found nominally significantly associated with cord blood telomere length, where the associations for the two SNPs in *OBFC1* gene were robust across coding methods and were of the same effect direction as the published GWAS’s (Prescott et al., 2011; Mangino et al., 2012; Codd et al., 2013; Codd et al., 2021). The *OBFC1* gene encodes the OB Fold-containing Protein 1, which is involved in telomere elongation (Wan et al., 2009). *KRT80*-encoded keratin 80 is involved in cell and tissue differentiation especially in epithelia (Langbein et al., 2010). Conditional on the SNP-CpG interaction, rs911847 (*SOD2*) was found strongly associated with cord blood telomere length, while in the discovery study it was associated with age at death and a skeletal marker of biological age (Lunetta et al., 2007). *SOD2* encodes a manganese ion-binding mitochondrial protein that converts superoxide byproducts of oxidative phosphorylation to hydrogen peroxide and oxygen. Deficiency of *SOD2* has been associated with premature epidermal thinning in mice, which is also an age-associated phenotype in human beings (Weyemi et al., 2012).

We assessed *cis*-mQTL for the candidate SNPs and all available nearby CpGs. Only *cis*-acting mQTL and interactions were investigated since the *trans*-interplay explains much less variation and tends to be polygenic (Gaunt et al., 2016). Part of the identified mQTLs in the current study were in line with an mQTL database published for a large-scale study of newborns (Gaunt et al., 2016) where the effect directions were also consistent. Only four SNPs, rs4452212 (*CXCR4*), rs11125529 (*ACYP2*), rs511744 (*SIRT3*) and rs17653722 (*KRT80*), were significantly associated with one CpG each, and all the other SNPs were associated with multiple CpGs. While rs3027234 (*CTC1*) was the mQTL with the largest number of CpGs, the SNP with the largest number of CpGs in the 500 kb neighborhood was rs40184 (*DAT1*). This might indicate that the presence of mQTL is not randomly distributed across the genome, but is enriched within a certain set of hub SNPs.

We found that DNA methylation at specific CpGs mediated the genetic association with newborn telomere length. All indirect effects were nominally significant, which is logical because the CpGs used to construct the mediation model were involved in mQTLs and associated with cord blood telomere length at the same time (meeting the minimal assumptions to be a mediator). None of the SNPs in the mediation model was associated with cord blood telomere length in the candidate gene association analysis, and neither were they when conditioned on the mediation by a CpG. Therefore, no SNP had a significant direct effect. Among the five SNP-CpG pairs used in the mediation analysis, rs2841505 (*SIRT5*)-cg01064902 (*SIRT5*) and rs412658 (*ZNF676*)-cg22620746 (*ZNF257*) confirmed the findings reported in mQTLdb. However, these SNP-CpG pairs have not been reported yet as being involved in a causal relationship with any diseases or phenotypes.

In addition to mediation, we also investigated effect modification by DNA methylation on the association between SNPs and cord blood telomere length. The variance explained by the model increased by 36% after adding the interaction between rs911847 (*SOD2*) and cg24223887 (gene unknown) (results not shown). Published epigenome-wide studies have reported DNA methylation modulating the genetic impact on cardiovascular disease-related traits (Veenstra et al., 2018; Wang et al., 2021b) and type II diabetes (Vohra et al., 2020), whereas for newborn telomere length, the current study is to our knowledge the first to identify SNP-CpG interactions.

In addition to the role of DNA methylation in the interplay with SNP genotypes that explained newborn telomere length, we previously have investigated DNA methylation through constructing a DNA methylation-based explanatory model for newborn telomere length (Wang et al., 2021a). However, we did not identify the same CpGs within an earlier reported DNAmTL prediction model (Lu et al., 2019), which was constructed using data from adult populations. Both telomere length and DNA methylation have highly dynamic patterns at birth as well as over life. Therefore, the epigenetic mechanisms of telomere length may be different throughout the stages of life.

Understanding genetic, epigenetic and environmental factors which can explain the variation of telomere length in early life is of interest as telomere length at birth is linked with telomere length in childhood and early adulthood (Martens et al., 2021). Moreover, telomere length at birth is a predictive factor for life expectancy (Heidinger et al., 2012) and has been reported to be associated with blood pressure in childhood (Martens et al., 2022). Our findings can potentially suggest clinical utility in prediction and intervention. The genotype at the SNPs under study might indicate different liability to telomere length shortening. While genotypes cannot be modified and can only be used for prognostic use, it is possible to alter the level of DNA methylation. For instance, the identified CpGs could possibly serve as targets in epigenetic therapies (Berdasco and Esteller, 2019). However, some identified CpGs were not mapped to any gene or with a known function, which requires further investigation for their biological or clinical relevance. Moreover, the current study is only exploratory and the findings still need validation in external populations in larger scale studies.

We acknowledge the limitations of the current study. Firstly, the placenta is a heterogeneous organ containing both fetal and maternal tissues. It is, however, unlikely that the SNPs were genotyped in maternal DNA, since standard sampling strategies and histological examinations was applied to minimize maternal tissue contamination, and studies have shown a largely differential molecular pattern between placental biopsies from different locations (Wyatt et al., 2005; Sood et al., 2006). Secondly, given the statistical power due to a small sample size and limited variation within a healthy population, we were not able to reproduce most of the associations between SNPs and telomere length that were identified in previous large-scale studies, which were mostly adult population-based. A haplotype-based approach that summarizes blocks of 10–100 kb might increase the power (Daly et al., 2001). However, since the SNPs included in the current study were selected *a priori* and were not from genome-wide genotyping, they only suggested SNP blocks of limited size (2–4 kb). Thirdly, the SNP selection was only based on genome-wide association studies, while it could be more precise and sensible to select targets based on genes that are well studied and known to be relevant for telomere length, such as telomerase or shelterin genes as suggested in the Telomerase Database (Podlevsky et al., 2008). In future investigations, such an improved target selection might further reveal important insights in the methylation mediating role of the genetic regulation of telomere biology. Fourthly, although indirect effects have been found *via* the DNA methylation at a few CpGs, the mediation effects should be interpreted with caution. The interplay between DNA methylation and telomere attrition is intertwined, because not only epigenetic modification but also telomere length can regulate gene expression (Kim and Shay, 2018), through which they might cause changes in each other. Further causal inference might be achieved by a Mendelian randomization study using genetic variants that are strongly associated with newborn telomere length as the instrumental variables (Davies et al., 2018). Finally, we acknowledge that the current study is hypothesis generating and that our identified markers should be further validated in larger follow-up studies and *via* using a targeted DNA methylation approach.

## 5 Conclusion

To conclude, our study for the first time modelled newborn telomere length in a context of multiple molecular layers (genetic variants and DNA methylation). We partly confirmed telomere length- or ageing-related genetic variants and mQTLs in newborns identified in previous studies. Our results suggest that DNA methylation might alter the effect of genetic variants on newborn telomere length, and identified DNA methylation loci that might be crucial targets in future investigations of telomere biology. Potential mediating effects by DNA methylation might be present, but knowledge of biological pathways is required for a sound causal inference. Through the current study, we have shown the potential importance of investigating the interplay between different molecular levels in regulating telomere length. This novel approach should be evaluated in future large-scale studies.

## Data Availability

The genetic variants frequency data is publicly available in the European Variation Archive with accession numbers PRJEB53351 (project) and ERZ11081188 (analyses). Other data are available from the corresponding author, DSM, upon reasonable request.
