# Supplementary material for: Genetic regulation of newborn telomere length is mediated and modified by DNA methylation
Source: Front Genet. 2022 Oct 4;13:934277. doi: 10.3389/fgene.2022.934277 (PMC9576874; doi:10.3389/fgene.2022.934277)
Supplement: Supplementary file 1 [file DataSheet1.docx]

**Supplementary materials**

**Genetic regulation of newborn telomere length is mediated and modified by DNA methylation**

Congrong Wang^1^, Rossella Alfano^1^, Brigitte Reimann^1^, Janneke Hogervorst^1^, Mariona Bustamante^2,3,4,5^, Immaculata De Vivo^6,7^, Michelle Plusquin^1^, Tim S. Nawrot^1,8^, Dries S. Martens^1^

^1^Centre for Environmental Sciences, Hasselt University, Hasselt, Belgium

^2^ISGlobal, Barcelona, Spain

^3^Universitat Pompeu Fabra, Barcelona, Spain.

^4^CIBER de Epidemiología y Salud Pública, Madrid, Spain

^5^Center for Genomic Regulation, Barcelona Institute of Science and Technology, Barcelona, Spain.

^6^Channing Division of Network Medicine, Department of Medicine, Brigham and Women’s Hospital and Harvard Medical School, Boston, MA 02215, USA

^7^Program in Genetic Epidemiology and Statistical Genetics, Harvard School of Public Health,

Boston, MA 02115, USA

^8^Department of Public Health & Primary Care, Leuven University, Leuven, Belgium

Supplementary Method 2

Telomere length measurement by qPCR assay 2

Supplementary Tables 3

Table S1. 26 SNPs related to telomere length and ageing involved in the current study. 3

Table S2. Numbers of SNP-CpG pairs in *cis* (±500kb) and the number of CpG that were significantly associated with each SNP. 4

Table S3. 57 SNP-CpG pairs identified. 5

Table S4. Associations between cord blood telomere length and the CpGs from the identified mQTL. 8

Table S5. Estimates from the mediation analysis. 9

Supplementary Figures 10

Figure S1. Sample inclusion criteria. 10

Figure S2. Statistical work flow in the current study. 11

Figure S3. Pairwise linkage disequilibrium (LD) in the 26 SNPs. 12

Figure S4. Relationship between SNP-CpG distance and the variance of CpG explained by SNP. 13

Supplementary Method

Telomere length measurement by qPCR assay

DNA samples were normalized to ensure a uniform DNA input of 5 ng for each qPCR, and this was checked using the Quant-iT™ PicoGreen® dsDNA Assay Kit (Life Technologies, Europe). All samples were measured in triplicate on a 7900HT Fast RealTime PCR System (Applied Biosystems) in a 384-well format. The telomere-specific qPCR reaction mixture contained 1x QuantiTect SYBR Green PCR master mix (Qiagen, Inc., Venlo, the Netherlands), 2 mM dithiothreitol (DTT), 300 nM telg primer (ACACTAAGGTTTGGGTTTGGGTTTGGGTTTGGGTTAGTG T) and 900 nM telc primer (TGTTAGGTATCCCTATCCCTATCCCTATCCCTATCCCTAACA). Used cycling conditions were: 1 cycle at 95°C for 10 min, 2 cycles at 94°C for 15 sec and 49°C for 2 min, and 30 cycles at 94°C for 15 sec, 62°C for 20 sec, and 74°C for 1 min and 40 sec. The single-copy gene (human β globin) qPCR mixture contained 1x QuantiTect SYBR Green PCR master mix, 400 nM HBG1 primer (GCTTCTGACACAACTGTGTTCACTAGC) and 400 nM HBG2 primer (CACCAACTTCATCCACGTTCACC). Used cycling conditions were: 1 cycle at 95°C for 10 min, 40 cycles at 95°C for 15 sec, and 58°C for 1 min and 20 sec. After each qPCR a melting curve analysis was performed. In each run, a 6-point serial dilution of pooled DNA was run to assess PCR efficiency. PCR-efficiencies ranged from 100-105% for telomere runs and 95-100% for single-copy gene runs. Furthermore 10 inter-run calibrators (IRCs) were run to account for inter-run variability over 16 qPCR plates. qPCR curves for each sample were visually inspected and when technical problems were detected or triplicates showed too high variability, samples were removed for further analysis. Telomeres were normalized using qBase (Biogazelle, Zwijnaarde, Belgium). The reliability of our assay was assessed by calculating the coefficient of variation (CV) within triplicates of the T/S ratios. In addition, the interclass coefficients (ICC) with 95% CI of triplicate measures of the T/S ratios,(1) both inter-assay (based on 10 IRCs over 16 qPCR plates) and intra-assay (based on all measures), were calculated using the available on-line R script on the Telomere Research Network website.(2)

**Reference**

1. TELOMERE RESEARCH NETWORK: Study Design & Analysis 2020 [Available from: <https://trn.tulane.edu/resources/study-design-analysis/>.

2. Eisenberg. D, Nettle. D, Verhulst. S. How to calculate the repeatability (ICC) of telomere length measures 2020 [Available from: <https://trn.tulane.edu/wp-content/uploads/sites/445/2020/10/How-to-calculate-repeatability.pdf>.

Supplementary Tables

Table S1. 26 SNPs related to telomere length and ageing involved in the current study.

| **SNP (major/minor allele)** | **Gene** | **Position** | **Discovery study** | **MAF^i^** | **HWE^ii^ p-value** |
| --- | --- | --- | --- | --- | --- |
| rs911847 (G/A) | *SOD2* | chr6:159647936 | Lunetta, K. L., et al. (2007) | 0.293 | 0.446 |
| rs1343981 (A/G) | *LEPR* | chr1:65579645 | Lunetta, K. L., et al. (2007) | 0.239 | 0.719 |
| rs1475398 (C/G) | *LEPR* | chr1:65517574 | Lunetta, K. L., et al. (2007) | 0.237 | 0.708 |
| rs2371208 (G/T) | *--* | chr7:82708543 | Lunetta, K. L., et al. (2007) | 0.233 | 0.644 |
| rs4764600 (C/G) | *GAPDH* | chr12:6492814 | Lunetta, K. L., et al. (2007) | 0.337 | 0.316 |
| rs6669117 (T/C) | *LEPR* | chr1:65595389 | Lunetta, K. L., et al. (2007) | 0.436 | 0.611 |
| rs10493379 (G/A) | *LEPR* | chr1:65580244 | Lunetta, K. L., et al. (2007) | 0.239 | 0.719 |
| rs10496799 (T/C) | *NXPH2* | chr2:139261401 | Lunetta, K. L., et al. (2007) | 0.281 | 0.062 |
| rs3757354 (C/T) | *MYLIP* | chr6:16235386 | Lunetta, K. L., et al. (2007) | 0.187 | 0.452 |
| rs4452212 (G/A) | *CXCR4* | Chr2:136258421 | Levy, D., et al. (2009) | 0.461 | 0.778 |
| rs16847897 (G/C) | *TERC* | chr3:169850328 | Prescott, J., et al. (2011) | 0.270 | 0.419 |
| rs412658 (C/T) | *ZNF676* | chr19:22176638 | Mangino, M., et al. (2012) | 0.330 | 0.296 |
| rs3027234 (C/T) | *CTC1* | chr17:8232774 | Mangino, M., et al. (2012) | 0.210 | 0.855 |
| rs9419958 (T/C) | *OBFC1* | chr10:103916188 | Mangino, M., et al. (2012) | 0.150 | 0.267 |
| rs755017 (A/G) | *RTEL1* | chr20:63790269 | Codd, V., et al. (2013) | 0.148 | 0.913 |
| rs9420907 (C/A) | *OBFC1* | chr10:103916707 | Codd, V., et al. (2013) | 0.157 | 0.179 |
| rs10936599 (C/T) | *TERC* | chr3:169774313 | Codd, V., et al. (2013) | 0.223 | 0.167 |
| rs11125529 (C/A) | *ACYP2* | chr2:54248729 | Codd, V., et al. (2013) | 0.143 | 0.384 |
| rs17653722 (G/T) | *KRT80* | chr12:52193734 | Liu, Y., et al. (2014) | 0.189 | 0.227 |
| rs40184 (T/C) | *DAT1* | chr5:1394962 | Mitchell, C., et al. (2014) | 0.481 | 0.529 |
| rs1386494 (T/C) | *TPH2* | chr12:71958763 | Mitchell, C., et al. (2014) | 0.138 | 0.492 |
| rs4570625 (G/T) | *TPH2* | chr12:71938143 | Mitchell, C., et al. (2014) | 0.205 | 0.133 |
| rs107251 (T/C) | *SIRT6* | chr19:4176088 | TenNapel, M. J., et al. (2014) | 0.092 | 0.663 |
| rs511744 (T/C) | *SIRT3* | chr11:219089 | TenNapel, M. J., et al. (2014) | 0.284 | 0.976 |
| rs2841505 (T/G) | *SIRT5* | chr6:13571363 | TenNapel, M. J., et al. (2014) | 0.318 | 0.123 |
| rs2535913 (G/A) | *DCAF4* | chr14:72948525 | Mangino, M., et al. (2015) | 0.281 | 0.433 |

^I^ MAF: minor allele frequency in the 281 samples used in the current study.

^ii^ HWE p-value: Hardy-Weinberg equilibrium chi-square test p-value in the 281 samples used in the current study.

Table S2. Numbers of SNP-CpG pairs in *cis* (±500kb) and the number of CpG that were significantly associated with each SNP.

| SNP | Gene name | SNP position | Number of  CpGs in *cis* | Number of associated CpGs for each SNP |
| --- | --- | --- | --- | --- |
| rs1475398 (C/G) | *LEPR* | chr1:65517574 | 349 | 2 |
| rs1343981 (A/G) | *LEPR* | chr1:65579645 | 348 | 3 |
| rs10493379 (G/A) | *LEPR* | chr1:65580244 | 348 | 3 |
| rs6669117 (T/C) | *LEPR* | chr1:65595389 | 346 | 2 |
| rs4452212 (G/A) | *CXCR4* | chr2:136258421 | 224 | 1 |
| rs10496799 (T/C) | *NXPH2* | chr2:139261401 | 73 | 0 |
| rs11125529 (C/A) | *ACYP2* | chr2:54248729 | 238 | 1 |
| rs10936599 (C/T) | *TERC* | chr3:169774313 | 414 | 6 |
| rs16847897 (G/C) | *TERC* | chr3:169850328 | 420 | 2 |
| rs40184 (T/C) | *DAT1* | chr5:1394962 | 1435 | 0 |
| rs2841505 (T/G) | *SIRT5* | chr6:13571363 | 372 | 4 |
| rs911847 (G/A) | *SOD2* | chr6:159647936 | 332 | 0 |
| rs3757354 (C/T) | *MYLIP* | chr6:16235386 | 294 | 0 |
| rs2371208 (G/T) |  | chr7:82708543 | 85 | 0 |
| rs9419958 (T/C) | *OBFC1* | chr10:103916188 | 680 | 0 |
| rs9420907 (C/A) | *OBFC1* | chr10:103916707 | 680 | 0 |
| rs511744 (T/C) | *SIRT3* | chr11:219089 | 952 | 1 |
| rs17653722 (G/T) | *KRT80* | chr12:52193734 | 686 | 1 |
| rs4764600 (C/G) | *GAPDH* | chr12:6492814 | 1014 | 9 |
| rs4570625 (G/T) | *TPH2* | chr12:71938143 | 237 | 0 |
| rs1386494 (T/C) | *TPH2* | chr12:71958763 | 243 | 0 |
| rs2535913 (G/A) | *DCAF4* | chr14:72948525 | 292 | 2 |
| rs3027234 (C/T) | *CTC1* | chr17:8232774 | 947 | 12 |
| rs412658 (C/T) | *ZNF676* | chr19:22176638 | 111 | 4 |
| rs107251 (T/C) | *SIRT6* | chr19:4176088 | 1074 | 4 |
| rs755017 (A/G) | *RTEL1* | chr20:63790269 | 0 | 0 |

Table S3. 57 SNP-CpG pairs identified significant (under Bonferroni correction) in both dominant coding and additive coding models, among which 22 mQTLs confirmed the findings in mQTLdb at birth.

| **SNP** | **SNP position** | **SNP gene** | **CpG** | **CpG position** | **CpG gene** | **binary model ^i^** | | **additive model** | |  | **mQTLdb** | |
| --- | --- | --- | --- | --- | --- | --- | --- | --- | --- | --- | --- | --- |
|  |  |  |  |  |  | **beta** | **adj. p-value** | **beta** | **adj. p-value** |  | **beta** | **p-value** |
| rs1475398 | chr1:65517574 | *LEPR* | cg05197142 | chr1:65990802 | *LEPR* | 0.268 | 5.44E-12 | 0.235 | 1.18E-14 |  |  |  |
| rs1475398 | chr1:65517574 | *LEPR* | cg06864398 | chr1:65774568 | *DNAJC6* | -0.150 | 1.09E-04 | -0.139 | 1.75E-06 |  |  |  |
| rs1343981 | chr1:65579645 | *LEPR* | cg05197142 | chr1:65990802 | *LEPR* | 0.278 | 2.87E-13 | 0.248 | 3.02E-16 |  |  |  |
| rs1343981 | chr1:65579645 | *LEPR* | cg09168320 | chr1:66031046 | *LEPR* | -0.391 | 1.20E-09 | -0.361 | 1.19E-12 |  |  |  |
| rs1343981 | chr1:65579645 | *LEPR* | cg06864398 | chr1:65774568 | *DNAJC6* | -0.175 | 4.59E-07 | -0.155 | 2.26E-08 |  |  |  |
| rs10493379 | chr1:65580244 | *LEPR* | cg05197142 | chr1:65990802 | *LEPR* | 0.275 | 6.30E-13 | 0.247 | 5.29E-16 |  |  |  |
| rs10493379 | chr1:65580244 | *LEPR* | cg09168320 | chr1:66031046 | *LEPR* | -0.398 | 4.10E-10 | -0.367 | 3.70E-13 |  |  |  |
| rs10493379 | chr1:65580244 | *LEPR* | cg06864398 | chr1:65774568 | *DNAJC6* | -0.173 | 7.16E-07 | -0.154 | 3.09E-08 |  |  |  |
| rs6669117 | chr1:65595389 | *LEPR* | cg09168320 | chr1:66031046 | *LEPR* | -0.572 | 5.23E-20 | -0.431 | 1.75E-29 |  |  |  |
| rs6669117 | chr1:65595389 | *LEPR* | cg05197142 | chr1:65990802 | *LEPR* | 0.224 | 7.08E-07 | 0.175 | 1.38E-10 |  |  |  |
| rs4452212 | chr2:136258421 | *CXCR4* | cg20242066 | chr2:136595261 | *LCT* | -0.267 | 2.73E-04 | -0.195 | 1.39E-07 |  |  |  |
| rs11125529 | chr2:54248729 | *ACYP2* | cg13006717 | chr2:54479214 | *ACYP2* | -0.213 | 2.66E-10 | -0.199 | 1.07E-10 |  | -0.709 | 9.67E-44 |
| rs10936599 | chr3:169774313 | *TERC* | cg08193579 | chr3:169529701 | *LRRC34* | 0.491 | 1.68E-04 | 0.452 | 2.84E-05 |  | 0.503 | 6.53E-24 |
| rs10936599 | chr3:169774313 | *TERC* | cg21687591 | chr3:169492002 | *MYNN* | -0.381 | 8.39E-20 | -0.333 | 9.96E-20 |  |  |  |
| rs10936599 | chr3:169774313 | *TERC* | cg13337095 | chr3:169531783 | *LRRC34* | 0.382 | 4.18E-05 | 0.358 | 4.53E-06 |  |  |  |
| rs10936599 | chr3:169774313 | *TERC* | cg04714994 | chr3:169530875 | *LRRC34* | 0.225 | 1.68E-04 | 0.208 | 2.84E-05 |  |  |  |
| rs10936599 | chr3:169774313 | *TERC* | cg03369965 | chr3:169531731 | *LRRC34* | 0.353 | 1.68E-04 | 0.336 | 2.84E-05 |  |  |  |
| rs10936599 | chr3:169774313 | *TERC* | cg05344026 | chr3:169530920 | *LRRC34* | 0.324 | 1.68E-04 | 0.302 | 2.84E-05 |  |  |  |
| rs16847897 | chr3:169850328 | *TERC* | cg08193579 | chr3:169529701 | *LRRC34* | 0.499 | 3.24E-04 | 0.465 | 5.19E-06 |  | 0.419 | 5.38E-17 |
| rs16847897 | chr3:169850328 | *TERC* | cg13337095 | chr3:169531783 | *LRRC34* | 0.338 | 6.27E-04 | 0.319 | 8.66E-06 |  |  |  |
| rs2841505 | chr6:13571363 | *SIRT5* | cg02206980 | chr6:13574034 | *SIRT5* | -0.468 | 3.38E-23 | -0.421 | 1.39E-32 |  | -0.626 | 1.33E-55 |
| rs2841505 | chr6:13571363 | *SIRT5* | cg01064902 | chr6:13574091 | *SIRT5* | 0.478 | 2.74E-20 | 0.392 | 3.59E-22 |  | 0.575 | 1.31E-33 |
| rs2841505 | chr6:13571363 | *SIRT5* | cg19366958 | chr6:13574150 | *SIRT5* | 0.216 | 1.79E-07 | 0.174 | 5.83E-08 |  | 0.308 | 1.14E-10 |
| rs2841505 | chr6:13571363 | *SIRT5* | cg07534331 | chr6:13574296 | *SIRT5* | 0.444 | 7.79E-05 | 0.446 | 2.63E-08 |  | 0.613 | 4.35E-54 |
| rs511744 | chr11:219089 | *SIRT3* | cg23513443 | chr11:236781 | *PSMD13; SIRT3* | -0.190 | 1.45E-07 | -0.161 | 3.19E-09 |  | -0.417 | 3.63E-19 |
| rs17653722 | chr12:52193734 | *KRT80* | cg08070646 | chr12:52616097 | *LOC283404* | -0.231 | 1.73E-06 | -0.195 | 1.45E-05 |  | -0.327 | 5.70E-09 |
| rs4764600 | chr12:6492814 | *GAPDH* | cg07142400 | chr12:6602447 | *NCAPD2; MRPL51* | 0.598 | 1.40E-32 | 0.522 | 1.15E-58 |  | 0.576 | 2.40E-52 |
| rs4764600 | chr12:6492814 | *GAPDH* | cg10692528 | chr12:6605071 | *NCAPD2* | 0.470 | 2.15E-19 | 0.363 | 6.79E-23 |  | 0.476 | 7.66E-24 |
| rs4764600 | chr12:6492814 | *GAPDH* | cg15719903 | chr12:6570167 | *TAPBPL* | 0.271 | 4.86E-11 | 0.220 | 9.41E-15 |  | 0.312 | 1.75E-16 |
| rs4764600 | chr12:6492814 | *GAPDH* | cg10383428 | chr12:6604190 | *NCAPD2* | 0.212 | 2.95E-08 | 0.181 | 3.41E-12 |  | 0.278 | 5.03E-09 |
| rs4764600 | chr12:6492814 | *GAPDH* | cg22129323 | chr12:6572482 | *VAMP1* | 0.706 | 4.67E-22 | 0.572 | 9.21E-30 |  |  |  |
| rs4764600 | chr12:6492814 | *GAPDH* | cg08283181 | chr12:6619112 | *NCAPD2; SCARNA10* | -0.196 | 3.40E-10 | -0.185 | 3.80E-19 |  |  |  |
| rs4764600 | chr12:6492814 | *GAPDH* | cg16399745 | chr12:6604039 | *NCAPD2* | 0.187 | 4.84E-10 | 0.147 | 2.84E-12 |  |  |  |
| rs4764600 | chr12:6492814 | *GAPDH* | cg25241256 | chr12:6580691 | *VAMP1* | 0.324 | 4.23E-09 | 0.257 | 2.52E-11 |  |  |  |
| rs4764600 | chr12:6492814 | *GAPDH* | cg05740381 | chr12:6630724 | *NCAPD2* | -0.204 | 3.37E-05 | -0.178 | 4.11E-08 |  |  |  |
| rs2535913 | chr14:72948525 | *DCAF4* | cg12610013 | chr14:73409314 | *DCAF4* | -0.250 | 6.91E-17 | -0.201 | 4.25E-19 |  |  |  |
| rs2535913 | chr14:72948525 | *DCAF4* | cg00792184 | chr14:73355148 | *DPF3* | -0.157 | 6.11E-05 | -0.121 | 5.61E-05 |  |  |  |
| rs3027234 | chr17:8232774 | *CTC1* | cg08322244 | chr17:8066669 | *VAMP2* | -0.889 | 3.61E-15 | -0.810 | 4.67E-18 |  | -0.469 | 3.04E-19 |
| rs3027234 | chr17:8232774 | *CTC1* | cg11257113 | chr17:8062204 |  | -0.266 | 5.95E-10 | -0.227 | 3.71E-10 |  | -0.293 | 2.94E-10 |
| rs3027234 | chr17:8232774 | *CTC1* | cg06726167 | chr17:8076949 | *TMEM107* | -0.203 | 8.72E-09 | -0.157 | 3.78E-07 |  | -0.336 | 8.46E-18 |
| rs3027234 | chr17:8232774 | *CTC1* | cg16597406 | chr17:8130173 | *C17orf68* | -0.349 | 5.01E-06 | -0.318 | 3.78E-07 |  | -0.336 | 1.58E-12 |
| rs3027234 | chr17:8232774 | *CTC1* | cg04219014 | chr17:8150601 | *CTC1* | 1.040 | 1.34E-28 | 0.887 | 3.11E-29 |  |  |  |
| rs3027234 | chr17:8232774 | *CTC1* | cg26173986 | chr17:8067016 | *VAMP2* | -0.542 | 2.56E-16 | -0.469 | 1.46E-17 |  |  |  |
| rs3027234 | chr17:8232774 | *CTC1* | cg22315544 | chr17:8066671 | *VAMP2* | -0.440 | 4.95E-16 | -0.398 | 9.42E-19 |  |  |  |
| rs3027234 | chr17:8232774 | *CTC1* | cg12459718 | chr17:8129997 | *C17orf68* | -0.509 | 1.08E-06 | -0.461 | 8.16E-08 |  |  |  |
| rs3027234 | chr17:8232774 | *CTC1* | cg14209583 | chr17:8214081 | *ARHGEF15* | -0.232 | 2.56E-06 | -0.205 | 4.55E-07 |  |  |  |
| rs3027234 | chr17:8232774 | *CTC1* | cg04055490 | chr17:8059012 |  | 0.083 | 4.13E-05 | 0.065 | 2.74E-04 |  |  |  |
| rs3027234 | chr17:8232774 | *CTC1* | cg19537511 | chr17:8213813 | *ARHGEF15* | -0.156 | 2.44E-04 | -0.138 | 9.73E-05 |  |  |  |
| rs3027234 | chr17:8232774 | *CTC1* | cg21143441 | chr17:8124011 | *C17orf44* | -0.128 | 3.19E-04 |  |  |  |  |  |
| rs412658 | chr19:22176638 | *ZNF676* | cg22620746 | chr19:22234992 | *ZNF257* | -0.648 | 4.41E-14 | -0.553 | 1.05E-17 |  | -0.683 | 5.88E-53 |
| rs412658 | chr19:22176638 | *ZNF676* | cg20662725 | chr19:22235022 | *ZNF257* | -0.364 | 1.88E-10 | -0.336 | 1.54E-15 |  | -0.573 | 1.20E-37 |
| rs412658 | chr19:22176638 | *ZNF676* | cg06394874 | chr19:22235850 | *ZNF257* | -0.494 | 3.95E-09 | -0.433 | 5.53E-12 |  | -0.477 | 2.00E-26 |
| rs412658 | chr19:22176638 | *ZNF676* | cg16672337 | chr19:22605111 | *ZNF98; ZNF98* | -0.308 | 4.79E-06 | -0.224 | 2.41E-05 |  |  |  |
| rs107251 | chr19:4176088 | *SIRT6* | cg23999422 | chr19:4173466 |  | 0.890 | 6.18E-24 | 0.814 | 3.84E-24 |  | 0.773 | 1.21E-39 |
| rs107251 | chr19:4176088 | *SIRT6* | cg09617135 | chr19:4173482 |  | 0.553 | 1.86E-16 | 0.498 | 6.16E-16 |  | 0.529 | 1.41E-18 |
| rs107251 | chr19:4176088 | *SIRT6* | cg23943923 | chr19:4194526 | *ANKRD24* | 0.172 | 1.35E-04 | 0.154 | 1.82E-04 |  |  |  |
| rs107251 | chr19:4176088 | *SIRT6* | cg02721952 | chr19:4182885 | *SIRT6; ANKRD24* | 0.245 | 1.35E-04 | 0.235 | 4.10E-05 |  |  |  |

All models were adjusted for newborn sex, gestational age, ethnicity, birth weight, maternal medical conditions during pregnancy, pre-pregnancy BMI, parity, education level, smoking status, paternal age and cell type heterogeneity.

Table S4. Nominally significant associations between cord blood telomere length and the CpGs from the identified mQTL (significance level of 5%).

| CpG | Position | Relation to Island | Gene | CpG regulatory feature | Estimate (p-value) |
| --- | --- | --- | --- | --- | --- |
| cg12610013 | Chr14:73409314 | Open Sea | *DCAF4* |  | 0.115 (p=0.043) |
| cg01064902 | Chr6:13574091 | N Shore | *SIRT5* | Promoter Associated | 0.055 (p=0.042) |
| cg14209583 | Chr17:8214081 | Open Sea | *ARHGEF15* |  | -0.080 (p=0.038) |
| cg22620746 | Chr19:22234992 | Open Sea | *ZNF257* | Promoter Associated | 0.045 (p=0.020) |
| cg22129323 | Chr12:6572482 | Open Sea | *VAMP1* | Promoter Associated | 0.046 (p=0.046) |

All models were adjusted for newborn sex, gestational age, ethnicity, birth weight, maternal medical conditions during pregnancy, pre-pregnancy BMI, parity, education level, smoking status, paternal age, and cell count heteroscedasticity.

Table S5. Estimates (bootstrap p-value) of direct, indirect and total effects from the mediation analysis.

| **SNP** | **Direct effect** | **mediator CpG** | **Indirect effect (p-value)** | **Total effect**  **(p-value)** |
| --- | --- | --- | --- | --- |
| rs2535913 (*DCAF4*) | -0.012 (p=0.63) | cg12610013 (*DCAF4*) | **-0.026(p=0.04)** | -0.038 (p=0.11) |
| rs2841505 (*SIRT5*) | -0.044 (p=0.13) | cg01064902 (*SIRT5*) | **0.039 (p=0.01)** | -0.0044 (p=0.82) |
| rs3027234 (*CTC1*) | -0.011 (p=0.65) | cg14209583 (*ARHGEF15*) | **0.020 (p=0.02)** | 0.0085 (p=0.72) |
| rs412658 (*ZNF676*) | 0.041 (p=0.14) | cg22620746 (*ZNF257*) | **-0.036 (p=0.01)** | 0.0047 (p=0.83) |
| rs4764600 (*GAPDH*) | -0.008 (p=0.80) | cg22129323 (*VAMP1*) | **0.040 (p=0.01)** | 0.032 (p=0.22) |

Estimates in bold were significant at 5% level. All models were adjusted for newborn sex, gestational age, ethnicity, birth weight, maternal medical conditions during pregnancy, pre-pregnancy BMI, parity, education level, smoking status, paternal age, and cell count heteroscedasticity.

Supplementary Figures

**
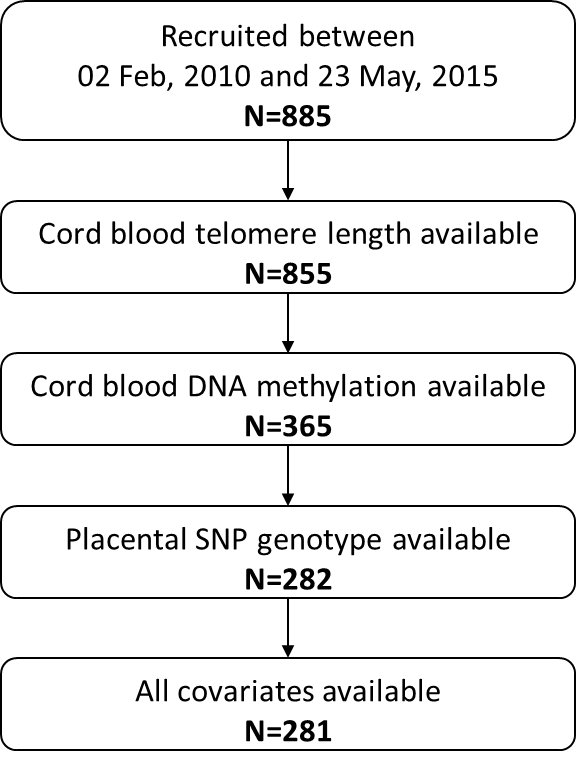
**

Figure S1. Sample inclusion criteria.

**
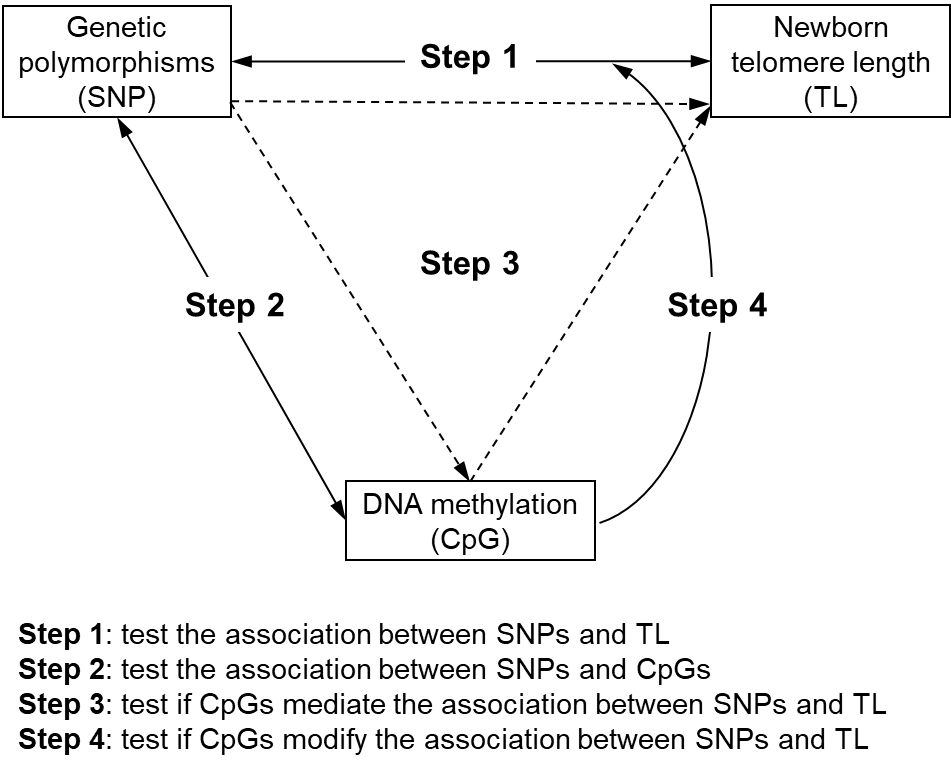
**

Figure S2. Statistical work flow in the current study.


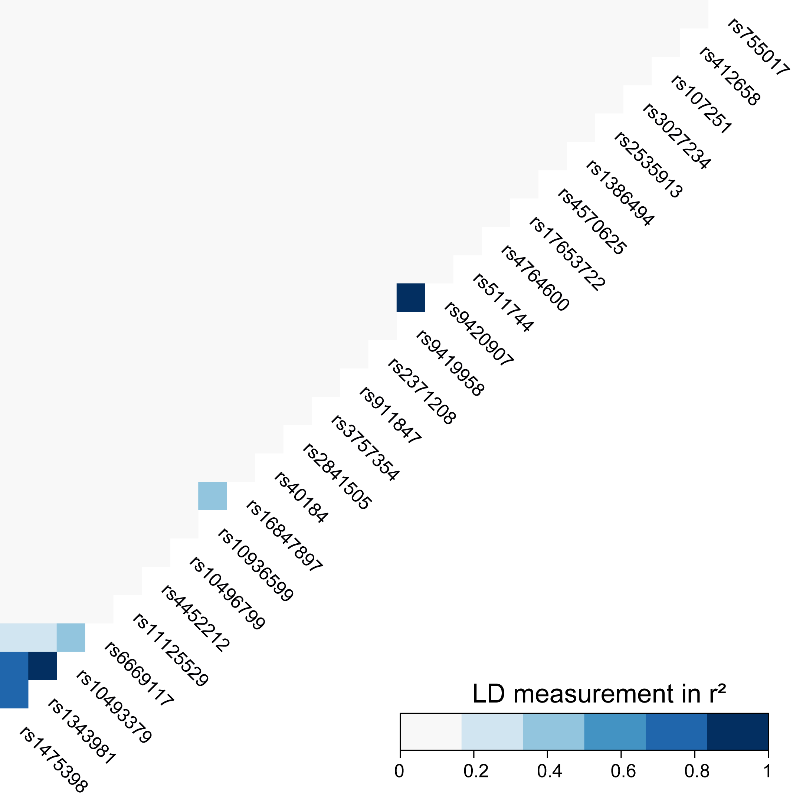


Figure S3. Pairwise linkage disequilibrium (LD) in the 26 SNPs. LD values were expressed in squared correlation coefficient r^2^.


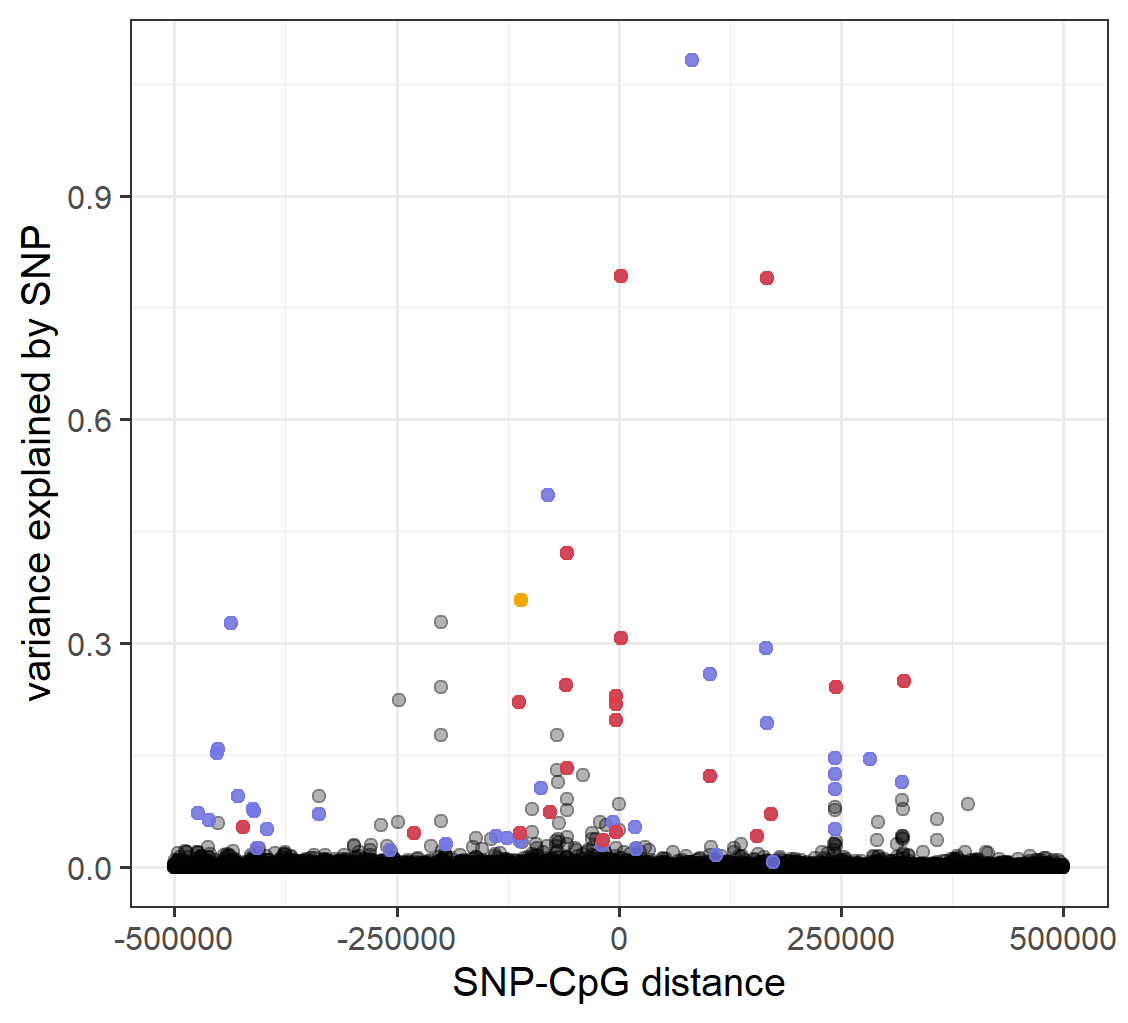


Figure S4. Relationship between SNP-CpG distance and the variance of CpG explained by SNP. Blue dots: 57 SNP-CpG pairs identified in the present study; red dots: 22 SNP-CpG pairs validating the external cis-mQTL database; yellow dot: the top signal SNP-CpG pair.
